# Evaluation of response to a cholera outbreak in January 2024 using the 7–1–7 timeliness metrics: a case study of Elegu Point of Entry, Uganda

**DOI:** 10.1186/s12889-024-20886-y

**Published:** 2024-12-04

**Authors:** Innocent Ssemanda, Brian Kibwika, Ritah Namusoosa, Benon Kwesiga, Lilian Bulage, Richard Migisha, Alex Riolexus Ario

**Affiliations:** Uganda Public Health Fellowship Program, Uganda National Institute of Public Health, Kampala, Uganda

**Keywords:** Cholera, Imported outbreak, Cross-border outbreak prevention and control, 7–1–7 metrics, Detection, Notification, Response, Uganda

## Abstract

**Background:**

Cholera is a major public health threat in Uganda, especially in border districts prone to outbreaks from cross-border movement. We investigated and evaluated the initial response to a January 2024 cholera outbreak in Elegu Town, on the Uganda-South Sudan border, using the 7–1–7 timeliness metrics to assess detection, notification, and response capacities, highlighting Uganda's preparedness and challenges in managing cross-border outbreaks.

**Methods:**

We defined a suspected case as the onset of acute watery diarrhea in an asylum seeker at the Elegu border point from January to February 2024. A confirmed case was a suspected case in which *Vibrio cholerae* was isolated in the stool by culture or PCR. We actively searched for cases and collected data on person characteristics, symptoms, and outbreak timeliness. We used semi-structured interviews to elicit insights from district health officials on the enabling factors and bottlenecks during the response. We used the 7–1–7 metric to assess detection, notification, and response capacities of the point of entry.

**Results:**

Thirteen members of a refugee family from South Sudan were diagnosed with cholera within 6 h of arrival at the Elegu border, with 4 (31%) confirmed cases. No death occurred. The authorities detected, notified, and responded to the outbreak within the 7–1–7 timelines, with no major bottlenecks identified. The outbreak was detected and notified within one day and by the fifth day, a full response was mounted. The prompt response was attributed to the availability of a functional emergency operations center and the presence of trained surveillance frontline health workers.

**Conclusion:**

Response to an imported cholera outbreak at Elegu border point demonstrated Uganda's preparedness in managing cross-border disease outbreaks. Achieving the 7–1–7 targets highlighted the country’s-built capacity to detect, notify, and respond to such events. Continued investment in local-level disease detection, communication, and national-level resource mobilization will be crucial to sustaining future effective cross-border outbreak prevention and control strategies.

**Supplementary Information:**

The online version contains supplementary material available at 10.1186/s12889-024-20886-y.

## Introduction

Cholera remains a global public health threat, causing between 1.3 million to 4.0 million cases, and 21,000 and 143,000 deaths worldwide every year [1, 2]. For > 50 years after its resurgence in Africa, cholera is still a major public health problem in Africa, accounting for an estimated 50–52.6% of the global cholera cases and 58.6–79.6% of the global cholera-related deaths annually [3]. Between 2010 and 2020, 25 African countries reported 484,450 suspected cholera cases and 999 cholera outbreaks to the World Health Organization (WHO) [4]. From the late 1990s through the first decade of the twenty-first century, sub-Saharan Africa has reported more cholera deaths than any other region in Africa. Between 2007 and 2011, the annual case fatality ratios (CFRs) for cholera in sub-Saharan Africa ranged from 2.2% to 3% [5–7]. This highlights the continued public health burden posed by cholera across sub-Saharan Africa, even in the face of advancements in scientific understanding and treatment of the disease.

In Uganda, epidemics of cholera have occurred regularly since the disease was first reported in 1971 and the disease has nearly become endemic, with cases reported every year since 2000 [8]. While many parts of the country have not experienced outbreaks of the disease, the border districts have had recurrent outbreaks in the last two decades [8, 9]. The Ministry of Health has instituted preventive and control measures that include the promotion of access to safe water, sanitation, and hygiene; health education and community mobilization; disease surveillance; and case management [10, 11]. However, cholera cases continue to be reported annually. Between 2011 and 2015, Uganda reported over 9,000 cases of cholera in 18 border districts, with an annual average of 60–182 deaths [9, 12]. In border districts, there is a greater chance of importation of cholera due to frequent travel by the communities across borders and also the influx of asylum seekers during conflicts in the neighboring countries [13]. Cholera poses a major threat to regions with vulnerable populations, such as refugees, fishing communities, and large urban slum settlements [9, 13].

Timely detection, reporting, and response to an infectious disease outbreak are critical to prevent localized health events from emerging as pandemic threats. Rapid detection depends on effective disease surveillance systems leveraging data from multiple sources [14–16]. Timeliness is a key criterion for evaluating any disease surveillance system. How fast a system can detect a threat is critical for ensuring optimal performance [14, 15]. Since the West Africa Ebola epidemic of 2014–16, several frameworks have been developed to measure readiness capacity. Uganda has adopted a new global target of 7–1–7 whereby every suspected outbreak is identified within 7 days of emergence, reported to public health authorities within one day, and effectively responded to within 7 days [15, 17]. With clear targets for each milestone, these metrics can inform real-time performance gaps including bottlenecks where targets are not being met. Building on the International Health Regulations (2005) and WHO’s “triple billion targets” methodology, 7–1–7 metrics simplify performance evaluation, provide a blueprint for outbreak communication, and drive performance improvement [18].

On January 21, 2024, the Ugandan Ministry of Health was alerted of 13 suspected cholera cases among south sudan asylum seekers who has just arrived at the refugee reception center at the Elegu Border Town, in Amuru District, bordering the Republic of South Sudan. The case patients presented with profuse vomiting and acute watery diarrhea. Four out of five stool samples from the patients tested positive for *Vibrio cholerae* by both rapid diagnostic test (RDT) and polymerase chain reaction (PCR). We investigated and evaluated the initial response to a cholera outbreak in Elegu Town, a border point between Uganda and South Sudan, in January 2024, highlighting the country's preparedness and challenges in responding to cross-border disease outbreaks using the 7–1–7 metric.

## Methods

### Study setting

The outbreak was reported at Elegu Point of Entry, which is located in Amuru District, bordering Nimule Town, South Sudan. Elegu Town has a population of 17,000 people. In 2016, this same area was affected by a cholera outbreak, with 44 (99%) of the cases being refugees from South Sudan [19]. In 2021, the Ministry of Health set up a Regional Public Health Emergency Operations Center in Arua to help coordinate response to public health emergencies in the West Nile Region. The refugees from the Elegu border refugee reception area are usually hosted in the neighboring Adjumani district which has both the refugee collection camp and settlement.

### Field investigation

We defined a suspected case as the onset of acute watery diarrhea in an asylum seeker at Elegu Town from January 16, 2024, to February 5, 2024. A confirmed case was a suspected case in which *Vibrio cholerae* was isolated in the stool by culture or PCR. At this point, we believed the outbreak was confined to the reception center for asylum seekers since there was no ongoing outbreak within the district, and we were aware of an outbreak occurring in South Sudan, the primary source of these refugees. However, we conducted an extensive active case search within the community and healthcare facilities, targeting both refugees and local residents to ensure a comprehensive assessment of the situation.

Using the case definition, we actively searched for cases in three of the most important health facilities and reception center communities in the border town. In the health facilities, we reviewed records and interviewed health workers, patients, and caretakers to gather information on potential cases of cholera. At the refugee reception and collection center, we interviewed new asylum seekers and refugee community leaders to obtain relevant information about the cholera outbreak. Since there was no ongoing outbreak in the district prior to the 13 cases, we did not conduct a district-wide active case search.

Using unstructured questions, we interviewed two health workers who received the patients upon their arrival: a clinician and a nurse. At the reception center health unit, we spoke with the facility in charge, selected patients and caretakers, and randomly sampled 10 asylum seekers who arrived on the same day as the case patients.

### 7–1–7 assessment

The 7–1–7 metric, which has been adopted by MoH, was proposed by WHO as a target for outbreak detection, notification, and early response, whereby every suspected outbreak is detected within 7 days of emergence and reported to public health authorities within 1 day of detection, and seven early response actions are completed within 7 days from reporting to public health authorities, indicating timely initiation of response [15]. We evaluated the team’s readiness to respond to public health emergencies using the 7–1–7 metrics (Table 1). We assessed the response timeliness, from the initial detection to the completion of the outbreak response activities. This start-to-end evaluation gauged the speed with which the district detected the outbreak, notified the MoH, and implemented the response measures. To gain insights into the response process, we still used the 7–1–7 to document the enabling factors and bottlenecks in the response.

**Table 1:** WHO’s 7–1–7 metrics for outbreak management evaluation

**Milestones**	**Date** DD/MM/YY	**Narrative** Briefly describe key observations in this interval and how the date was determined
**Date of emergence** *For endemic diseases:* the date on which a predetermined increase in case incidence over baseline rates occurred *For non-endemic diseases:* the date on which the index case or first epidemiologically linked case first experienced symptoms *For other public health events:* date the threat first met criteria as a reportable event based on country reporting standards		
**Date of detection** Date the event is first recorded by any source or in any system		
**Date of notification** Date the event is first reported to a public health authority responsible for action		
**Date of early response initiation** Date on which the first of the seven early response actions occurred (see below) 1. Initiate investigation or deploy investigation/response team 2. Conduct epidemiologic analysis of burden, severity, and risk factors, and perform initial risk assessment 3. Obtain laboratory confirmation of the outbreak etiology 4. Initiate appropriate case management and infection prevention and control (IPC) measures in health facilities 5. Initiate appropriate public health countermeasures in affected communities 6. Initiate appropriate risk communication and community engagement activities 7. Establish a coordination mechanism		

### Data analysis

We performed a descriptive analysis of the personal characteristics of the cases, presenting numerical findings as frequencies and percentages. For the qualitative data from the 7–1–7 documented enablers and bottlenecks, we carefully reviewed the information to ensure the accuracy and appropriate representation of the participants' perspectives, as demonstrated by the inclusion of direct quotations in the manuscript.

## Results

### Characteristics of case-patients, cholera outbreak at Elegu, Uganda-South Sudan border, January 2024

This was a cholera outbreak among 13 asylum seekers who belonged to a family of 14 members. Originally from Khartoum, Sudan, they first sought refuge in the town of Ruweng at the border between Sudan and South Sudan. They later moved to Elegu Town where they presented with symptoms. On January 21, 2024, three hours after their arrival, 13 of the 14 family members developed acute watery diarrhea and vomiting, which are symptoms of cholera. They sought care at a nearby private facility within two hours of symptom onset. All thirteen (100%) case-patients had both diarrhea and vomiting. Eleven (85%) had general body weakness and 6(46%) had abdominal pain (Table 2).

**Table 2:** Characteristics of case-patients, Elegu, Uganda-South Sudan border, January 2024, (*n* = 13)

Characteristic	n (%)
**Sex**	
Male	4 (31)
Female	9 (69)
**Age**	
Child (< 18)	7 (54)
Adult (≥ 18)	6 (46)
**Case Status**	
Confirmed	4 (31)
Suspect	9 (69)
**Symptoms**	
Diarrhea	13 (100)
Vomiting	13 (100)
Body weakness	11 (85)
Abdominal pain	6 (46)

When the index case presented symptoms, followed shortly by other family members, they sought care at the facility adjacent to the refugee reception and collection center. The clinician immediately suspected cholera due to the presentation of loose stools and vomiting. Samples were collected shortly thereafter, and treatment was initiated without delay. Stool samples from five of the 13 suspects were sent to the Uganda National Health Laboratories, Kampala. Four out of the five (80%) samples tested positive for cholera on both RDT and PCR. Of the 13 individuals, seven (54%) were children, and nine (69%) were female.

### Time characteristics of the cholera outbreak at Elegu, Uganda-South Sudan border, January 2024

**Fig. 1:**
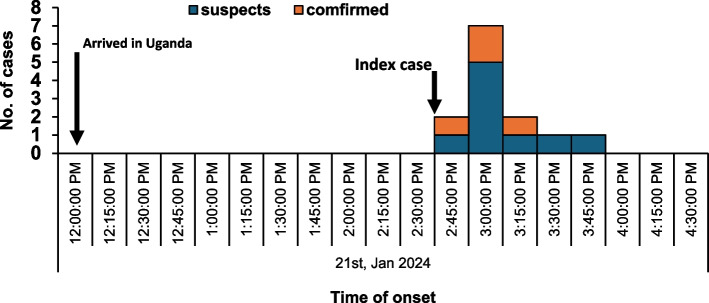
Distribution of cases by time of onset of symptoms at Elegu, Uganda-South Sudan border, January 2024 (*n* = 13)

The first case-patient presented with symptoms upon arrival in Uganda, two and a half hours later. In less than one and a half hours, all the 13 case-patients had presented with symptoms (Fig. 1).

### Timeline of the cholera outbreak at Elegu, Uganda-South Sudan border, January 2024

**Fig. 2:**
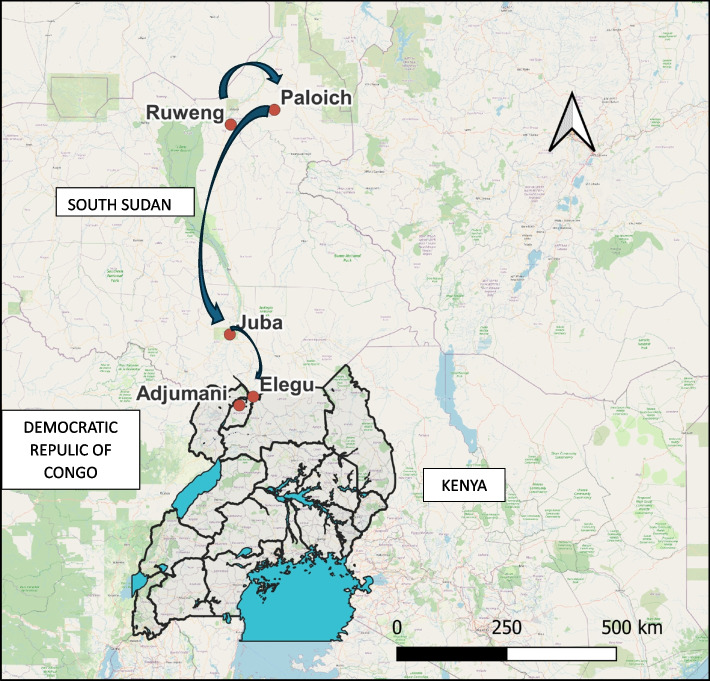
Affected Family's Journey: From Ruweng, South Sudan to Elegu, Uganda, January 2024

The family had been staying in the Ruweng Town Refugee Settlement in South Sudan for a month, planning to travel to Uganda. On January 17, 2024, they left the settlement and headed south, reaching the city of Paloich on the night of January 18, 2024. They then booked a flight to Juba two days later, on January 20, 2024 (Fig. 2).

**Fig. 3:**
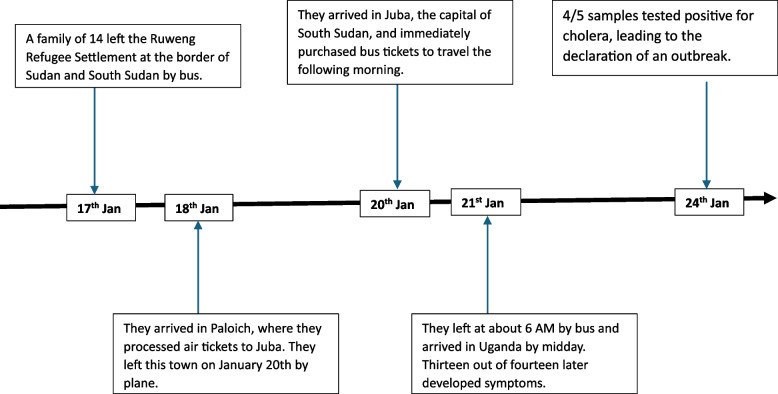
A timeline of the family travel history to the cholera outbreak declaration at Elegu, Uganda-South Sudan border, January 2024

While in Ruweng, the family bought food and other groceries from street vendors within the refugee settlement and used water from the settlement reservoir, which was treated with chlorine. The settlement had a high influx of refugees from Sudan and an ongoing cholera outbreak at the time. The family reported receiving cholera vaccines in Sudan three months prior, but there was no evidence to corroborate this. On their journey to Paloich, the family ate a local bread called Tamia, along with water they had packed. In Paloich, they bought rice and fish from the local market and used water from the reservoir, which was provided and treated by an NGO. Once in Juba, the family bought eggs, meat, water, and powdered juice from a supermarket, which they cooked and consumed. The next morning, they packed some eggs and bread but did not eat anything until they crossed the border into Uganda at the Nimule-Elegu border point. Three hours after the last meal, 13 of the 14 members fell ill as summarized in Fig. 3 below.

### 7–1–7 assessment findings, cholera outbreak at Elegu, Uganda-South Sudan border, January 2024

#### Detection

The initial cholera outbreak was detected on January 21, 2024, at the Elegu Point of Entry (PoE). A cluster of 14 asylum seekers sought clearance at the PoE health desk, and the index case, a 15-month-old male, exhibited initial cholera-like symptoms. The refugee reception center authorities suspected cholera and promptly completed a Case Investigation Form (CIF) on the same day. According to interviews with district officials who led the response and the frontline health workers, several key factors enabled this timely detection:

Adjumani is a border district, and border districts in Uganda are the most affected by cholera outbreaks due to importation. The district was aware of the situation across the border, and all health workers, both in the private and public sectors, had recently participated in continuing medical education (CME) on cholera. Information, Education, and Communication (IEC) materials had been distributed, and the district remains on high alert due to its unique position.

"The availability of appropriate case definitions and guidelines allowed us to quickly recognize the symptoms and suspect cholera," noted the District Surveillance Focal Person (DSFP).

"The presence of a screening facility at the Elegu Refugee Collection and Reception Center was crucial for identifying these cases early" commented a Nyumanzi Refugee Settlement official.

“The swift detection of the outbreak was facilitated by the good attitude and ethics of the health workers. Our prior experience in dealing with cross-border issues during the COVID-19 pandemic also played a role in the timely response" added Assistant District Health Officer (ADHO).

#### Notification

Immediately following the detection, the surveillance team from Adjumani District took swift action. An alert was sent using the 6767 system to the electronic Integrated Disease Surveillance and Response (eIDSR) platform. Additionally, a phone call was made to Medical Teams International (MTI), an NGO supporting refugee settlements in the region, requesting Emergency Medical Services (EMS) for the suspected cases. Interviews with the surveillance team revealed that several key factors enabled this timely and effective response:

"Our knowledge of the existing alert management system, including the 6767 platform and eIDSR, allowed us to respond promptly" stated a member of the surveillance team.

"Availability of the necessary tools and resources, such as case definitions and guidelines, ensured we had the right information to act on the alert" remarked another team member.

"The clear communication and reporting channels from the Elegu Refugee Reception Center to the district-level surveillance team facilitated the rapid notification during the outbreak" added the DSFP.

#### Response

The cholera outbreak was responded to by a combined team of members from Adjumani District, Arua PHEOC, and Elegu Port Health personnel. The response to the potential cholera outbreak was initiated immediately on the same day the index case was detected at the Elegu Point of Entry (PoE). The District Rapid Response Team (DRRT) was swiftly deployed to investigate the situation, collect laboratory specimens, and initiate treatment while evacuating the suspected cases.

“Some of the response team members have recently had training in frontline field epidemiology, these were very resourceful as we mounted the response,” said the District Health Officer (DHO).

The outbreak was detected, and authorities were notified within a single day. Furthermore, by day 5 of the outbreak, all response pillars had been functionalized (Table 3).

**Table 3:** Calculated timelines within the 7–1–7 period, cholera outbreak at Elegu, Uganda-South Sudan border, January 2024

**Interval**	**Calculation** In days	**Timeliness** In days	**Target** In days	**Met target?** Yes/No
**Detection**	Difference between dates of emergence and detection	**1**	**7**	**YES**
**Notification**	Difference between dates of detection and notification	**1**	**1**	**YES**
**Response**	Difference between dates of notification and completion of the last early response action	**5**	**7**	**YES**

## Discussion

We investigated an imported cholera outbreak in Uganda and evaluated the initial response, highlighting the preparedness and challenges associated with cross-border outbreak prevention and control in the country. Thirteen asylum seekers traveling from South Sudan to Uganda were diagnosed with cholera, 6 h after they arrived at the Elegu POE. The authorities were able to detect, notify, and respond to the outbreak within the stipulated timelines as per the 7–1–7 metric, and no significant bottlenecks were identified in the response to this outbreak.

Our findings indicate that the case-patients were exposed to cholera during their stay in Ruweng Town, most likely on the day of departure since it marks the first day among the 5 days of incubation. The outbreak in Ruweng was reportedly imported from Sudan, which had experienced a cholera outbreak since September 2023, with approximately 10,000 cases and more than 290 deaths [20, 21]. Cholera importation is not uncommon, as shown by several studies [22, 23]. Ugandan districts near the border have been at the highest risk of such cholera outbreaks. A study on cholera surveillance in Uganda found that cholera was persistently occurring in the northwestern border districts of the country [6]. If feasible, all asylum seekers from regions with ongoing cholera outbreaks should be screened and vaccinated at the border points to reduce the risk of importation. The border point is equipped with rapid diagnostic tests, and the country has four laboratories capable of conducting PCR and culture sensitivity tests. Additionally, the country, through its partners, has the capacity to deploy vaccines when deemed necessary.

This assessment highlights the vulnerability of asylum seekers specifically and immigrants in general to cholera and other waterborne diseases during their displacement and migration. A study on cholera prevention and control in refugee settings found that these outbreaks consistently involved inadequate water chlorination, a lack of sanitation facilities, and improper disposal of cholera waste [24]. Refugees may encounter different strains of cholera or other pathogens along their journey, which may require different prevention and treatment strategies. Therefore, it is essential to provide adequate water, sanitation, and hygiene (WASH) facilities, health education, and oral cholera vaccine (OCV) to asylum seekers and other displaced populations, especially in areas with endemic or epidemic cholera [24, 25].

In this study, we describe the use of the 7–1–7 metric for reporting on the timeliness of outbreak response, which was designed to align with and support the implementation of the International Health Regulations (IHR), specifically, the capacities at the community or primary public health response level, intermediate public health response level, and national level [15, 18]. We found that for the imported cholera outbreak at Elegu, the Uganda-South Sudan border point in January 2024, the authorities were able to detect, notify, and respond to the outbreak within the stipulated 7–1–7 timelines. Prompt response initiation was observed, with the outbreak being detected and notified within 1 day. However, it's also important to note that this was a relatively smaller outbreak containing a few cases without secondary cases, which could have facilitated this exceptional performance.

According to the 7–1–7 metric, the authorities responding to the outbreak, met the targets for detection, notification, and reporting [15]. These were done in 1:1:5 days, compared to the set target of 7:1:7 days respectively [17]. This performance can be attributed to the recent experience in responding to Ebola and COVID-19 outbreaks. The outbreak found the district with an existing and functional Regional Emergency Operations Centre (REOC), trained frontline health workers, and a response structure with clear pillars and terms of reference [26].

The fact that the frontline health workers at the district and the reception center had had some training in surveillance show that disease detection capacities must continue to be developed at both public and private health facilities, as most events are detected by health workers outside the public health system. A study done in Uganda comparing both private and public facilities capacity to detect disease outbreaks further highlighted this need [27]. Clear communication and reporting channels between health workers and surveillance officers are crucial to verifying events and initiating a larger public health response [17]. The analysis revealed that the most frequent response bottlenecks such as resource limitations and the availability of countermeasures, at the district level were not significant issues during this outbreak. However, the national-level and partner support resources to augment these gaps were a major enabling factor in the response.

These insights underscore the importance of strengthening disease detection and response capacity at the local level while ensuring effective coordination and resource mobilization at the national level to support a comprehensive and timely outbreak management strategy. This investigation indicates that the 7–1–7 target is achievable during outbreak management and highlights the importance of continued system strengthening.

## Study limitations

A key limitation of this study is the lack of corroborating findings from the neighboring countries of South Sudan and Sudan. Investigating cross-border outbreaks requires a coordinated, regional approach, and without data from these settings, the transferability of the lessons learned may be constrained. Future investigations would benefit from increased collaboration and data-sharing between Uganda and surrounding countries to strengthen the evidence base for managing infectious disease threats at shared borders. Secondly, the reliance on qualitative interview data is a key limitation of this assessment, as information gathered through interviews can be subject to potential information biases. Lastly, this is a single case study of a successful outbreak detection and response in a small area with limited actors and among a single family. It may not be representative of the broader situation in the country as a whole.

## Conclusion

Despite the challenges posed by the outbreak among asylum seekers from South Sudan, Uganda demonstrated effective control by meeting the 7–1–7 targets for detection, notification, and response. The prompt detection, notification, and response were facilitated by the availability of a functional emergency operations center and trained frontline health workers. However, the study also highlights the need for enhanced collaboration and data-sharing with neighboring countries to strengthen the evidence base for managing infectious disease threats at shared borders. Moving forward, it is essential to continue strengthening disease surveillance and response systems in affected areas, including refugee collection centers and border points. Regular screening and mass vaccination campaigns with oral cholera vaccine should be conducted to prevent and control cholera among vulnerable populations. These interventions, along with ongoing efforts to improve cross-border collaboration, will be crucial in enhancing Uganda's preparedness and response to future cross-border outbreaks.

## Supplementary Information

Supplementary Material 1.

## Data Availability

No datasets were generated or analysed during the current study.

## Abbreviations

CFR: Case-Fatality Ratio
CPHL: Central Public Health Laboratories
CIF: Case Investigation Form
eIDSR: Electronic Integrated Disease Surveillance and Response
EMS: Emergency Medical Services
IHR: International Health Regulations
RCCE: Risk Communication and Community Engagement
REOC: Regional Emergency Operations Centre
RDT: Rapid Diagnostic Test
DRRT: District Rapid Response Team
WASH: Water, Sanitation, and Hygiene
OCV: Oral Cholera Vaccine
PCR: Polymerase Chain Reaction
PoE: Point of Entry
DTF: District Task Force
US CDC: United States Centers for Disease Control and Prevention
