# Supplementary material for: Evaluation of response to a cholera outbreak in January 2024 using the 7–1–7 timeliness metrics: a case study of Elegu Point of Entry, Uganda
Source: BMC Public Health. 2024 Dec 4;24:3370. doi: 10.1186/s12889-024-20886-y (PMC11616103; doi:10.1186/s12889-024-20886-y)
Supplement: Supplementary file 1 — Supplementary Material 1. [file 12889_2024_20886_MOESM1_ESM.docx]

Annex1:

**Cholera outbreak, Adjumani district, January 2024.**

**Case Investigation form**

Hello, my name is ...................... I am a health worker from the Ministry of Health. In January 2024, there was an Outbreak of Cholera in Adjumani which is still going on. We are visiting homes in this village to interview people to try and find out how and why the outbreak came up and spread to it and prevent similar outbreaks in the future. Your answers shall be kept confidential. You shall not receive any direct benefits, but your honest answers will help in ling this outbreak.

| Do you agree to participate? (Yes/No) | If No, end the interview there |
| --- | --- |
| Date of interview |  |
| Name of interviewer |  |
| A. CASE IDENTIFICATION | |
| 1. Coordinates of the household location |  |
| 2. Case ID |  |
| 3. Household ID |  |
| 4. Name of respondent |  |
| 5. Contact of respondent |  |
| 6. Relationship of respondent to the case or |  |
| 7. Name of Case |  |
| B. SOCIO-DEMOGRAPHIC CHARACTERISTICS OF THE CASE | |
| 1. Age of the Case Patient in years |  |
| 2. Sex |  |
| 3. Occupation |  |
| 4. Religion |  |
| 5. Level of education |  |
| 6. Tribe |  |
| 7. Name of Case patient Village |  |
| 8. Name of Case patient parish |  |
| 9. Name of Case patient Subcounty |  |
| 10. Name of Case patient District |  |
| 11. How many people normally sleep in this household? |  |
| D. SIGNS AND SYMPTOMS | |
| 1. Which signs and symptoms did the case patient get? |  |
| 2. What was the date of onset of first symptom? |  |
| 3. What was the time of onset of first symptom |  |
| 4. Did the case patient seek care? |  |
| 5. Where did the case patient seek care from? |  |
| 6. Name of the facility place where the case patient first sought care |  |
| 7. When did the case patient first seek care? |  |
| 8. What was the treatment given |  |
| 10. Was there any problem obtaining treatment? |  |
| 11. Was the case patient admitted? |  |
| 12. What was the date of outcome (discharge or death)? |  |
| 13. Was any test for cholera done? |  |
| 14. If yes, which test was done? |  |
| E. EXPOSURES | |
| 1. What is the case patient’s source(s) of drinking water? |  |
| 2. What is the case patient’s source(s) of water for domestic use? |  |
| 3. Did you share a meal with someone who had an acute diarrheal illness? |  |
| 4. Did anyone in your household have acute diarrheal illness? |  |
| 5. From January 15th 2024 to-date, where have you washed your clothes from? |  |
| 6. From January 15th 2024 to-date, where have you bathed from? |  |
| 7. Did you have a latrine? |  |
| 8. From January 15th 2024 to-date, where were you defecating? |  |
| 9. Did anyone else in your household fall ill with acute diarrheal illness? |  |
| D. Travel History | |
| From January 15th 2024 to-date, Did you travel to anywhere? |  |
| On your journey, did you drink any thing? |  |
| What did you drink? |  |
| What was the source of the drink that you took? |  |
| On your journey, did you eat any thing? |  |
| What did you eat? |  |
| What was the source of the food/bite that you ate? |  |
| Where you traveled, where there any people suffering from diarrheal disease? |  |
